# Capacity building for implementation research: a methodology for advancing health research and practice

**DOI:** 10.1186/s12961-020-00568-y

**Published:** 2020-06-01

**Authors:** Phyllis Dako-Gyeke, Emmanuel Asampong, Edwin Afari, Pascal Launois, Mercy Ackumey, Kwabena Opoku-Mensah, Samuel Dery, Patricia Akweongo, Justice Nonvignon, Moses Aikins

**Affiliations:** 1 Department of Social and Behavioural Sciences, School of Public Health, University of Ghana, Geneva, Switzerland; 2 Department of Epidemiology and Disease Control, School of Public Health, University of Ghana, World Health Organization, Geneva, Switzerland; 3 World Health Organization, Geneva, Switzerland; 4 Department of Biostatistics and Health Informatics, School of Public Health, University of Ghana, Accra, Ghana; 5 Department of Health Policy, Planning and Management, School of Public Health, University of Ghana, Accra, Ghana

**Keywords:** Implementation research, capacity-building, LMICs, Africa, practitioners

## Abstract

**Background:**

Implementation research is increasingly being recognised as an important discipline seeking to maximise the benefits of evidence-based interventions. Although capacity-building efforts are ongoing, there has been limited attention on the contextual and health system peculiarities in low- and middle-income countries. Moreover, given the challenges encountered during the implementation of health interventions, the field of implementation research requires a creative attempt to build expertise for health researchers and practitioners simultaneously. With support from the Special Programme for Research and Training in Tropical Diseases, we have developed an implementation research short course that targets both researchers and practitioners. This paper seeks to explain the course development processes and report on training evaluations, highlighting its relevance for inter-institutional and inter-regional capacity strengthening.

**Methods:**

The development of the implementation research course curriculum was categorised into four phases, namely the formation of a core curriculum development team, course content development, internal reviews and pilot, and external reviews and evaluations. Five modules were developed covering Introduction to implementation research, Methods in implementation research, Ethics and quality management in implementation research, Community and stakeholder engagement, and Dissemination in implementation research. Course evaluations were conducted using developed tools measuring participants’ reactions and learning.

**Results:**

From 2016 to 2018, the IR curriculum has been used to train a total of 165 researchers and practitioners predominantly from African countries, the majority of whom are males (57%) and researchers/academics (79.4%). Participants generally gave positive ratings (e.g. integration of concepts) for their reactions to the training. Under ‘learnings’, participants indicated improvement in their knowledge in areas such as identification of implementation research problems and questions.

**Conclusion:**

The approach for training both researchers and practitioners offers a dynamic opportunity for the acquisition and sharing of knowledge for both categories of learners. This approach was crucial in demonstrating a key characteristic of implementation research (e.g. multidisciplinary) practically evident during the training sessions. Using such a model to effectively train participants from various low- and middle-income countries shows the opportunities this training curriculum offers as a capacity-building tool.

Contributions to the literature

- Identified processes used in developing an implementation research (IR) training module for low- and middle-income countries using a multi-disciplinary team.
- The contribution of a stepwise approach to developing, piloting and rolling out IR training to health researchers and practitioners emphasising the best approaches to ensuring successful health interventions.
- The realisation of the usefulness of IR capacity-building and knowledge-sharing that takes place when researchers and practitioners sit together in training.

## Background

Implementation research (IR) is a growing field promoting a successful response to the complexities encountered when implementing evidence-based health interventions. The discipline of IR is increasingly recognised as an important academic function for maximising the health benefits of interventions. Consequently, there are several capacity-building initiatives focusing either on mentorship, the development of key competencies and reporting guidelines, or on training on ethical issues [1–6]. Although these attempts are noteworthy, several have been developed for high-income settings, with very limited attention to the contextual and health system peculiarities in low- and middle-income (LMICs) countries [7]. Evidently, successful transfer of evidence-based interventions into practice is dependent on contextual factors [8]. Moreover, IR is multidisciplinary in nature and pivots around leads given by practitioners regarding the challenges encountered during implementation. Although many areas of science (e.g. basic research) do not require engagement from stakeholders, IR necessarily calls for engagement with practitioners [9]. This attempt is not only to enhance practitioner readiness but also to maximise the likelihood that research informs practice and for the needs of practitioners related to required evidence, available resources and means for sustainability to be taken into consideration [9]. Consequently, this growing the field requires a creative attempt to ensure the building of expertise for both health researchers and practitioners. It also means that the capacity of practitioners must be strengthened alongside that of health researchers to produce collaborative effort for the ultimate adoption and adaptation of health interventions.

There are considerable difficulties to overcome here. Although the health service sector has the potential to be a context for carrying out high-quality IR, there is a lack of a clear set of research competencies that is coupled with the slow pace of capacity development [10–12]. In order to address this, there should be a training model that initiates partnerships between practitioners who might have little research skills and researchers who may lack practice experiences [13, 14]. Both categories of professionals should be granted the opportunity to continuously reflect on the realities of health research through a team-based approach and play roles to ensure the successful implementation of health interventions [15].

Under its strategic focus, the WHO’s Special Programme for Research and Training in Tropical Diseases (TDR) promotes capacity-building in good health research practices globally [16, 17]. Through these efforts, the WHO-TDR initiated the establishment of Regional Training Centres in all WHO Regions (The African Regional Training Centre in Ghana for the African Region, Astana Medical University in Kazakhstan for the European Region, Research Institute for Tropical Medicine in Philippines for the Western Pacific Region,

Institut Pasteur de Tunis in Tunisia for The Eastern Mediterranean Region, Centro Internacional de Entrenamiento e Investigaciones Médicas (CIDEIM) in Colombia for the Americas, Universitas Gadjah Mada in Indonesia for the South East Asian Region). TDR supports this network of Regional Training Centres (RTCs), one of which has been selected on a competitive basis to conduct and disseminate training courses in IR. In October 2014, the University of Ghana School of Public Health (UGSPH) was selected as the WHO-TDR African Regional Training Centre to lead capacity-building in the area of IR. In this regard, in 2015, UGSPH began the development of an IR training model, which targets both health researchers and practitioners within LMIC contexts. This paper aims at demonstrating the Principles of IR (PIR) curriculum and course development processes. We also use feedback from course evaluations to highlight its relevance, not only for inter-institutional capacity-building but also for inter-regional networking and capacity strengthening.

## Methods and processes

**Fig. 1:**
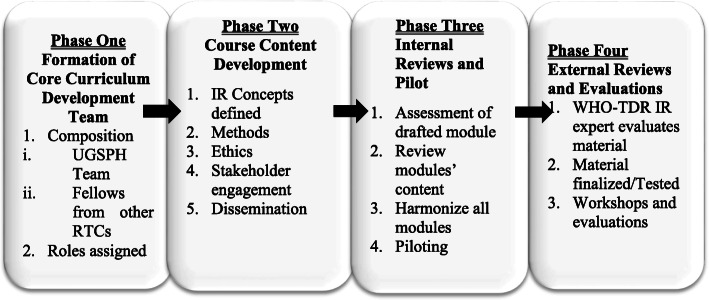
Course development phases

The development of the PIR curriculum followed a logical and systematic method that informed selected sets of activities. Key learnings from Thomas et al. [18] guided us through sets of activities, which we have isolated into four different phases as shown in Fig. 1.

These phases were informed by a needs assessment conducted through various searches (desk-top reviews, reading reports) and consultations (talking to faculty and Departmental Heads) seeking to identify existing institutionalised IR capacity-building efforts within LMICs. Among the very few that were identified in the sub-region was the MSc Applied Health Social Science programme currently run by the Department of Social and Behavioral Sciences, UGSPH, with a core focus on IR.

### Phase one: formation of core curriculum development team

The course development commenced with the formation of a core team mandated to lead the processes (Appendix 2). This team comprised of faculty from the UGSPH, who are involved in IR in various capacities, e.g. course development, teaching, grant application writing, reviewing and conducting IR (Table 1). This team was later expanded to include fellows from other RTCs, supported by WHO-TDR. Through meetings, research and consultations, the core team determined the scope of this short course (i.e. course objectives, core competencies, target audience, duration, course and module descriptions). Of critical importance, the team established the need to employ a pedagogic approach that would allow for the training of academics and health researchers as well as public health practitioners.

**Table 1:** Principles of Implementation Research (IR) curriculum content

Modules	Title	Description
Module 1	Introduction to IR	Unit 1: Concepts in IR
		• Scope of IR and its relevance
		Unit 2: Needs assessment for IR
		▪ IR problem and strategy identification
		▪ Theories and frameworks in IR
Module 2	Methods in IR	Unit 1: Formulating IR problems, questions and objectives
		Unit 2: Common research approaches in IR ▪ Quantitative methods
		▪ Qualitative methods
		▪ Mixed methods
Module 3	Ethics and Quality Management in IR	Unit 1: Ethics in IR
		• Key ethical principles in public health
		• Ethical issues in IR
		Unit 2: Quality management IR
		• Quality assurance
		• Quality management, etc.
Module 4	Stakeholder and Community Engagement in IR	Unit 1: Stakeholder engagement
		▪ Identifying stakeholders
		▪ Stakeholder Engagement
		Unit 2: The community in IR
		▪ What is a community?
		▪ Types of communities in IR
Module 5	Dissemination and Scale-Up in IR	Unit 1: Dissemination
		▪ Communication elements
		▪ Dissemination strategies and tools
		Unit 2: Barriers/facilitators of scaling-up: ▪ Producing and using evidence
		▪ Scaling up, types and elements ▪ References

### Phase two: course content development

The second phase consisted of sets of activities, which focused on the development of course content. Initial suggestions by the team regarding the scope of the short course were submitted to TDR for inputs and confirmation, following which five modules were defined (i.e. concepts, methods, ethics, stakeholder engagement and dissemination). After this scoping process, a series of meetings, consultations, research, discussions and presentations were held between March and September 2015. These processes were uniquely strategized to focus on institutional capacity-building for all WHO/TDR RTCs. Consequently, from July 2015 to September 2015, fellows from the various RTCs with TDR sponsorship came to Ghana and were involved in content development with mentorship from UGSPH faculty. Two fellows from the Research Institute for

Tropical Medicine, Philippines, were assigned Module 2; one fellow from Universitas Gadjah Mada in Indonesia and two fellows from Institut Pasteur de Tunis in Tunisia worked on Modules 1 and 5, respectively. A fellow from Astana Medical University, Kazakhstan, was assigned to work on Module 3. Finally, two fellows from the CIDEIM in Colombia were assigned Module 4. This mentoring mechanism included assignment of modules, discussion of learning objectives, presentations, etc. The presentations provided the opportunity for faculty to make inputs where necessary. Fellows also had the opportunity to contact faculty on a one-to-one basis for assistance. This was a unique opportunity to develop a network on IR capacity-building effort for both faculty and fellows.

### Phase three: internal reviews and pilots

Activities in the first two stages led to the development of a draft version of Modules 1–5. At this stage, the UGSPH team (Appendix 1) was paired and assigned to conduct an internal review of the Zero draft. With the use of a template, internal reviewers were to assess consistency in learning objectives, the relevance of selected subtopics, overlaps in content, the duration assigned and other areas of interest. After the internal reviews, a retreat was organised on 5–7 October 2015 at Aburi, Eastern Region, Ghana. This was to create a bigger forum to engage all content developers (i.e. UGSPH/RTC and Fellows), external capacity-building experts and some observers (i.e. TDR Representatives). At this meeting, fellows made presentations and received comments from reviewers. All participants then agreed on where additional work needed to be done. After all revisions were completed, the first pilot was conducted.

### Phase four: external reviews and evaluations

Following the first pilot, the entire curriculum was submitted to an anonymous external reviewer, through WHO-TDR. In April 2017, the external reviewer’s report was received and an internal meeting held on 10 May 2017 to study and address recommendations (e.g. fine-tuning the pedagogic approach to ensure relevance for both researchers and practitioners). Additionally, there was the need to include a fieldwork component to allow for practical application of IR concepts.

#### PIR course curriculum overview

The IR course curriculum is taught during organised workshops often taken face-to-face, usually within 4 days. The curriculum is organised in three sessions on each of the days intersected with snack and lunch breaks. Participants are trained using the five-module course developed. Each module is organised and delivered in units. The courses are presented by different facilitators during the indoor session of the workshop. Participants are put into groups on a daily basis to deliver presentations on assigned activities with the aim of building competencies for team/group work and presentations. A day’s field work component has been incorporated in accordance to the suggestions by the external reviewer. The fieldwork component involves participants and facilitators visiting Health Directorates (e.g. Decision-making institutions, Departments/Agencies etc.), non-governmental organisations in health, communities, and any such institution that may be undertaking relevant and applicable health intervention in real-life contexts. This is a carefully planned activity, undertaken after the modules on Introduction, Methods, Ethics and Community engagement have been delivered within the classroom setting, to help participants connect the understanding and knowledge acquired in the classroom with the pragmatic application of the practice of public health on the ground.

Generally, the content of the curriculum was structured with the objective of building/strengthening IR capacity among health researchers and practitioners within LMICs. Specifically, the course focuses on enhancing competencies in IR conceptualisation, design, successful execution of IR studies, stakeholder engagement, dissemination and scale-up of the IR strategies. Expected outcomes of the IR curriculum are to strengthen capacity of practitioners and researchers; to solve implementation problems observed in real-life situations, recognise key ethical issues and maximise engagement of key stakeholders at all processes of IR execution and dissemination of findings for better uptake.

#### Overview of courses run

From 2016 to 2018, qualified applicants were invited to participate in workshops for the PIR training. The course used varied teaching techniques (e.g. practical activities, lectures, class discussions, site visits, group work, etc.). All learning sessions were followed by participant evaluations. Module and workshop evaluations were employed in evaluating the curriculum. Data collection was carried out after each module in the case for ‘module evaluation’ and after the workshop for ‘workshop evaluation’. Participation in the evaluation was voluntary and questionnaires were made non-identifiable to ensure confidentiality.

PIR courses taken in 2016 and 2017 (i.e. pilot stages of the curriculum development) were assessed using open-ended questions exploring participants’ perceptions on sequence of topics presented in the modules, time allocation for presentations and activities as well as knowledge/skills acquired. Again, questions eliciting general comments and recommendations for improving the training material were obtained from trainees. The aim was to enable participants to provide detailed feedback on content and mode of delivery. Responses from these evaluations served as the basis for improving the curriculum developed.

In 2018, a more structured evaluation tool entailing close ended and semi-structured items was designed based on two criteria on Kirkpatrick’s framework for evaluation [19]. In totality, the framework assumes four criteria (reaction, learning, behavioural changes and organisation) often used in immediate and long-term training evaluations [20, 21]. For the purposes of this paper, we report on two of these criteria (reaction and learning), which were adequate in assessing the immediate impact of the PIR course on health practitioners and researchers. We use data from the structured tool to report on participants’ ‘reaction’ to the training programme. The evaluation questions included items for measuring both the content of modules as well as the entire workshop quality. The assessment tool consisted of nine-items on a 5-point Likert ordinal scale compiled by adapting questions from the Kirkpatrick model-based survey. Modal scores were determined for each of the items. For ‘learnings’, we used information from the semi-structured section of the questionnaire, which focuses on the knowledge and skills acquired during this training.

## Results

The PIR course was developed to provide competencies in the conceptualisation and design of IR studies, application of ethical principles, engagement of appropriate stakeholders as well as dissemination and scale-up. Thus far, the PIR workshop has been organised at least once in four countries (Ghana, Mozambique, Colombia and Jamaica). The results present the demographic characteristics of trainees as well as participants’ evaluations of the effectiveness of the IR curriculum.

### Demographic characteristics of participants

From 2016 to 2018, the PIR curriculum has been used to train 165 participants across the world. The majority of the participants were men (57%), researchers/academics (79.4%) and with Masters Level of education (50.3%). At the inception of the training programme in 2016, 17 men and 18 women were enrolled; the number increased to 30 men and 31 women in 2017 and made room for more researchers/academics and practitioners/policy-makers to participate in the PIR course. Furthermore, with four different workshops organised in 2018, the course recorded the highest attendance (*n* = 69) in a single year. Overall, more researchers and practitioners from African countries have been trained compared to other non-African countries (Table 2).

**Table 2:** Demographic characteristics of Principles of Implementation Research (PIR) participants

PIR participants (*n* = 165) Total	Years			
	2016 (*n* = 35)	2017 (*n* = 61)	2018 (*n* = 69)	
Gender				
Men	17	30	47	94 (57.0%)
Women	18	31	22	71 (43.0%)
Position				
Practitioners/Policy-makers	1	18	15	34 (20.6%)
Researchers/Academics	34	43	54	131 (79.4%)
Educational Qualification				
Bachelor	–	2	17	19 (11.5%)
Masters	28	26	29	83 (50.3%)
PhD	7	33	23	63 (38.2%)
Nationality				
Ghana	11	25	39	75 (45.5%)
Mozambique	–	2	24	26 (15.8%)
Jamaica	–	11	–	11 (6.7%)
Nigeria	4	3	1	8 (4.8%)
Colombia	2	9	–	11 (6.7%)
Sierra Leone	3	3	–	6 (3.6%)
Mali	2	1	–	3 (1.8%)
Malawi	2	–	–	2 (1.2%)
Kenya	2	–	–	2 (1.2%)
Rwanda	2	–	1	3 (1.8%)
Other African nationalities	4	3	4	11 (6.7%)
Others	3	4	–	7 (4.2%)

### Participants’ reactions to PIR training

Using a 5-point Likert scale, participants’ reactions to the PIR training in 2018 were assessed. The majority of the participants had positive reactions to the course (Table 3). Items evaluated included that the facilitator provided an opportunity for practice and contribution to discussions, the facilitator integrated the concepts, time allocation, understanding of the course and content usefulness. Participants’ reaction to PIR training was excellent, with rated modal scores of 5 for seven out of nine items measuring reaction to the course and mode of delivery (Table 3).

**Table 3:** Evaluation of Facilitator by Participants’ Reaction to Principles of Implementation Research Short course

Evaluated Items	Researchers	Implementers
Provided opportunity	5	5
Integrated concepts	5	5
Explained concepts	5	5
Spoke clearly	5	5
Time	5	5
Understanding	5	5
Content useful	5	5
Total average score	5	5

### Evaluation of overall course delivery

We also evaluated the reaction to the extent to which PIR course objectives and participants’ expectations for enrolment were met. Each of these items were rated with a modal score of 5, indicating that their expectations were totally met. Again, satisfaction with group work, the workshop as well as whether trainees will recommend the PIR course to others, were assessed. Group work sessions incorporated into training programmes were also evaluated as excellent. Again, trainees were positive with regards to overall satisfaction with the PIR workshop. This therefore was an indicator of their decision to recommend the PIR course to others, which was rated as excellent with a modal score of 5 (Table 4).

**Table 4:** Evaluation of overall course delivery

Course delivery	Average scores
PIR objectives met	5
Expectation met	5
Group work	5
Workshop	5
Recommend PIR course to others	5
Total	5

### Evaluation of participants’ learning

The evaluation of the learning was mainly obtained through responses to open-ended questions on the semi-structured tool, which focused on acquisition of knowledge and skills. The findings indicated that, prior to the training, some participants did not have much understanding of what IR was but gained knowledge on this during the training. For instance, a participant mentioned that “*I learnt and probably mastered the actual meaning of IR. Before now, it was really hazy*” (Participant 3, June 2016 PIR workshop). Another participant mentioned that: “*This is an eye opener in my study as a student. I did not actually get the understanding of what IR was but now I have in-depth understanding of IR*” (Participant 2, May 2017 PIR workshop). For those participants who had come across IR in their fields of practice or research earlier also received a clearer explanation of what IR was, its concepts and the characteristics of IR. This is evident in the quotes below:

“*Most of the issues that I read about in the literature about IR have been clarified and addressed.*” (Participant 4, June 2016 PIR workshop)

More importantly, participants learned about the research pipeline and the differences between IR and other types of research and how they are each situated on the health research pipeline. The distinction between IR and other streams of research served as the basis acquired by all individuals who participated in PIR workshops. For instance, some participants stated that:

“*I can now differentiate implementation research from other types of research such as operational research or health system research*.” (Participant 18, June 2016 PIR workshop)

“*I like this module* [Introduction to Implementation Research]*. It gives a good concept on implementation research. It actually makes the difference between IR and other researches. Implementation research deals with intervention.*” (Participant 11, June 2016 PIR workshop)

In addition to the basic IR concepts, some participants also mentioned that they had gained knowledge on ethical principles and quality control. The training gave participants an understanding on ethical considerations, quality control in IR, their significance as well as the distinction between these concepts. For instance, participants said:

“*I now know the difference between quality assurance and quality control.*” (Participant 5, May 2017 PIR workshop)

“*I learnt about the importance of Ethics and quality management when doing implementation research.*” (Participant 3, May 2017 PIR workshop)

Coupled with the theoretical knowledge acquired by participants, the workshops also provided skills. According to the trainees, the PIR workshop had offered them skills on different study designs and data collection approaches relevant in IR. For instance, some participant stated:

“*I have also learnt how to competently design/plan an IR which I previously did not have the skills to do before this training.*” (Participant 3, June 2016 PIR workshop)

“*Module two of the PIR course was very insightful for me. I am a purely quantitative researcher, but I have now been introduced to qualitative and mixed methods.*” (Participant 4, June 2016 PIR workshop)

“*I acquired knowledge and skills on overcoming barriers in scaling up innovations.*” (Participant 20, June 2016 PIR workshop)

Another practical learning was on how to identify relevant stakeholders using an analysis tool. Participants appreciated the significance of the involvement of community and stakeholders in an IR programme. One trainee was of the view that “*The success of any intervention is dependent on the support and participation of the community in which the intervention is implemented, thus the need to identify and prioritise ones stakeholders*” (Participant 2, June 2016 PIR workshop). In essence, the skills required to identify and engage key stakeholders in an IR programme were also learnt. This is evident in the following quote:

“*I have a better understanding of community mobilisation, social mobilisation and stakeholder engagement.*” (Participant 1, May 2017 PIR workshop)

## Discussion

Herein, we outlined the processes for the development of a training curriculum for the PIR short course with the aim of enhancing IR capacity. Our approach of providing training for both implementers/practitioners and researchers concurrently enhanced the incorporation of an intersectoral and interinstitutional collaboration. The overall result from the assessment of participants who took the developed PIR course indicated (1) consistency of the developed PIR course with stated lesson objectives, (2) relevance of the course content to both practitioners and researchers, and (3) consistency in sequence and linkages of PIR modules.

In disseminating the course development procedure, it is therefore essential to highlight key learnings revealed through the development and utilisation of this PIR curriculum.

### Learnings from a collaborative training model

The multidisciplinary nature of IR was evident through the use of the collaborative training model, which innovatively included both practitioners and researchers in training sessions. To achieve this aim, an elaborative consultative approach was utilised in enhancing interest during the development stage as well as to ensure participation by these specific stakeholders [22, 23]. Despite its usefulness, there were a few challenges that are noteworthy. During several sessions, we observed gaps in knowledge. Whereas researchers were a more advanced in their thinking of research principles, there was the need to spend more time with practitioners to enable them to better appreciate IR concepts. On the other hand, practitioners had depth of knowledge on the practical challenges that are encountered in the field, especially their understanding of how contexts impact the implementation processes. Consequently, there were concerns with content in terms of how much to offer within the sessions and time in terms of total training duration. In addressing these issues, we introduced a fieldwork component, combined some modules and reduced the number of days for training from 5 to 4 days. All these were targeted to enhance learning and facilitate the transfer of knowledge of the PIR in real life situations [24–26]. These help to overcome the potential of inert knowledge problems experienced in cases where expected transfer does not take place due to factors such as low turnover of stakeholders and challenges in embedding new programmes into existing systems [24, 27].

One of the spin-offs from this process was the increased opportunities for fellows from the different RTCs to interact and to share professional experiences. Stakeholders were invited to comment, either in a written format or through small workshops, on the drafted curriculum frameworks and the detailed content. Some stakeholders with particular expertise were invited to give guest lectures to students during the pilot. An important result from this enhanced process of interaction was the strengthening of an emerging IR training network as previous findings on the relevance of social networking and multi-stakeholder engagements in project success also indicated [23, 28–30].

### Implications for interregional/institutional capacity-building

The curriculum focused on sustainable outcomes for interregional and interinstitutional capacity-building and dissemination of the IR Concept. This was first evident in the approach of including fellows from various RTCs in various LMICs (Colombia, Indonesia, Tunisia and Philippines). Secondly, the initial opportunities to run the course were in these countries. We used this opportunity to further identify relevant case studies that will allow the course to be run globally in LMICs. Adoption of the corresponding expected changes in IR design and practices as well as training of researchers/practitioners have become the primary responsibility of universities and health institutions across cultures [31–34]. Since health practitioners, lecturers and researchers come from different backgrounds and traditional research trainings [35], it is necessary to create more internal awareness and capacity in the institutes. The curriculum development process and its implementation are means of capacity-building in the institutions. The knowledge obtained on concepts of IR, research methods, ethics, quality assurance, communication and community engagement will enhance healthcare delivery and good clinical outcomes.

### Limitation of the curriculum

The processes for curriculum development were intensive and the outcome has impacted positively on participants’ knowledge and skills. Participants generally gave positive ratings (e.g. integration of concepts) for their reactions to the training. Under ‘learnings’, participants indicated improvement in their knowledge in areas such as identification of IR problems and questions. However, there a few limitations. First, there is a language barrier, since all materials are in English language. In instances where training has taken place in Spanish- and Portuguese-speaking countries, it has been difficult for us to determine if indeed learning has taken place. Second, since we use only Neglected Tropical Diseases as examples in all the training sessions, some participants are limited in their understanding of IR concepts, especially individuals who work in other disease and public health areas.

## Conclusion

In this IR curriculum, we adopted an approach for training both researchers and implementers, which offers a dynamic opportunity for the acquisition and sharing of knowledge for both categories of learners. This approach was crucial in demonstrating the ability to make a key characteristic of IR (e.g. multidisciplinary) practically evident during the training sessions. Using such a model to effectively train participants from various LMICs shows the opportunities this training curriculum offers as a capacity-building tool.

### Supplementary information

**Additional file 1.**

## Data Availability

All relevant data are within the paper. The evaluation forms are also available on request.

## Appendix 1

Reviewers of drafted curriculum and modules assigned

- Module 1: PDG and FA
- Module 2: EA1 and AY
- Module 3: MA and JN
- Module 4: EA2 and OM
- Module 5: AA and PA

## Appendix 2

**Table 5:** Stakeholder engagements

Stakeholders	Functions and contributions	Institutions
Stakeholders inside training		
Curriculum developers	Designing curriculum	UGSPH, RTCs, etc.
Subject lectures	Teaching/participate in review	
University Heads of Department	Managing/monitoring	
Students	Evaluating training	
Stakeholders outside training		
Researchers	Consulting the content/participating in teaching	UGSPH, RTCs
Policy-makers	Participating in curriculum design	MOH, GHS
Health project managers (rural/urban)	Participating in training needs assessment/curriculum design	MOH, GHS
Training managers at ministerial level Control	Control/approvalParticipating in teaching material development	UGSPH, RTCs
Researchers/fellows	Participating in training needs assessmentConsulting/participating in training course design, signing training contracts	UGSPH, RTCs
INGO, LNGO	Consulting/participating in training course design, signing training contracts	WHO, TDR
Sponsor linking	Linking, coordinating activities	
Patients	Participating in research activities	GHS
Graduate students	Monitor/evaluating training courses	Universities

*GHS* Ghana Health Service, *INGO* international non-governmental organisations, *LNGO* local non-governmental organisation, *MOH* Ministry of Health, *RTCs* regional training centres, *TDR* Research and Training in Tropical Diseases, *UGSPH* University of Ghana School of Public Health

## Abbreviations

CIDEIM: Centro Internacional de Entrenamiento e Investigaciones Médicas
IR: Implementation research
LMICs: Low- and middle-income countries
PIR: Principles of implementation research
RTCs: Regional Training Centres
TDR: Research and Training in Tropical Diseases
UGSPH: University of Ghana School of Public Health
