# Supplementary material for: Capacity building for implementation research: a methodology for advancing health research and practice
Source: Health Res Policy Syst. 2020 Jun 1;18:53. doi: 10.1186/s12961-020-00568-y (PMC7268492; doi:10.1186/s12961-020-00568-y)
Supplement: Supplementary file 1 — Additional file 1. [file 12961_2020_568_MOESM1_ESM.docx]

# **Additional file 1**

**AFRICAN REGIONAL TRAINING CENTRE SUPPORTED BY WHO/TDR**

**WHO Africa Regional Training Center**

**University of Ghana, School of Public Health**

**Instructor and Module Evaluation**

**Date:_________________________**

**Instructor:_____________________________ Module_____________________**

**Circle the number that best describes how the instructor and module content met the following statements:**

| **The above named instructor :** | **Poor**  **Average** **Excellent** |
| --- | --- |
| 1. Provided opportunities for participants to practice, receive and give feedback. | 1 2 3 4 5 |
| 1. Integrated concepts with practice | 1 2 3 4 5 |
| 1. Explained concepts clearly and understandably | 1 2 3 4 5 |
| 1. Spoke and responded to participant questions clearly and thoroughly. | 1 2 3 4 5 |
| **This Module Content** |  |
| 1. The time allocated to this module was sufficient | 1 2 3 4 5 |
| 1. Rate the level of understanding of this module. | 1 2 3 4 5 |
| 1. Will the content of this module be useful to you? | 1 2 3 4 5 |
| 1. Did the activities help you to understand the information presented? | 1 2 3 4 5 |
| 1. Did this module meet its learning Objectives? | 1 2 3 4 5 |

Please comment on any other aspect of the instructor or module content that will be helpful:

______________________________________________________________________

______________________________________________________________________

______________________________________________________________________

FORM A-02

**WHO Africa Regional Training Center**

**University of Ghana, School of Public Health**

**End of Workshop Evaluation**

**Date: _________________________**

**Please circle the number that best describes how you rate the quality of this Workshop. Please add comments to help us improve future Workshops.**

|  | **Never**  **Sometimes** **Always** |
| --- | --- |
| 1. Were the learning objectives communicated clearly? | 1 2 3 4 5 |
| 1. Did the quality of the workshop content meet   your expectations? | 1 2 3 4 5 |
| 1. Was the venue conducive to your learning needs? | 1 2 3 4 5 |
| 1. Would you recommend this workshop to others? | 1 2 3 4 5 |
| **Rate the aspect of the workshop *(Please circle corresponding to your rating).*** | |
|  | **Poor**  **Average** **Excellent** |
| 1. Please rate the workshop as a whole | 1 2 3 4 5 |
| 1. Group work /exercise | 1 2 3 4 5 |
| 1. Meals | 1 2 3 4 5 |

1. What is your overall opinion of this Workshop?

________________________________________________________________

________________________________________________________________

1. What did we do well?

________________________________________________________________

________________________________________________________________

1. What can we do better or differently to improve this workshop?

________________________________________________________________

________________________________________________________________

1. Additional Comments:

______________________________________
